# A Polyhydroxybutyrate (PHB)-Biochar Reactor for the Adsorption and Biodegradation of Trichloroethylene: Design and Startup Phase

**DOI:** 10.3390/bioengineering9050192

**Published:** 2022-04-28

**Authors:** Marta M. Rossi, Sara Alfano, Neda Amanat, Fabiano Andreini, Laura Lorini, Andrea Martinelli, Marco Petrangeli Papini

**Affiliations:** 1 Department of Chemistry, Sapienza University of Rome, Piazzale Aldo Moro 5, 00185 Rome, Italy; sara.alfano@uniroma1.it (S.A.); neda.amanat@uniroma1.it (N.A.); laura.lorini@uniroma1.it (L.L.); andrea.martinelli@uniroma1.it (A.M.); marco.petrangelipapini@uniroma1.it (M.P.P.); 2 Ecotherm s.r.l., Via Vaccareccia, 43/D, 00071 Rome, Italy; fabiano.andreini@ecothermspa.it

**Keywords:** bioremediation, biochar, *Dehalococcoides*, reductive dechlorination, polyhydroxy butyrate

## Abstract

In this work, polyhydroxy butyrate (PHB) and biochar from pine wood (PWB) are used in a mini-pilot scale biological reactor (11.3 L of geometric volume) for trichloroethylene (TCE) removal (80 mgTCE/day and 6 L/day of flow rate). The PHB-biochar reactor was realized with two sequential reactive areas to simulate a multi-reactive permeable barrier. The PHB acts as an electron donor source in the first “fermentative” area. First, the thermogravimetric (TGA) and differential scanning calorimetry (DSC) analyses were performed. The PHB-powder and pellets have different purity (96% and 93% *w*/*w*) and thermal properties. These characteristics may affect the biodegradability of the biopolymer. In the second reactive zone, the PWB works as a *Dehalococcoides* support and adsorption material since its affinity for chlorinated compounds and the positive effect of the “coupled adsorption and biodegradation” process has been already verified. A specific dechlorinating enriched culture has been inoculated in the PWB zone to realize a coupled adsorption and biodegradation process. Organic acids were revealed since the beginning of the test, and during the monitoring period the reductive dichlorination anaerobic pathway was observed in the first zone; no chlorinated compounds were detected in the effluent thanks to the PWB adsorption capacity.

## 1. Introduction

Environmental issues associated with chlorinated aliphatic hydrocarbons (CAHs), e.g., chlorinated solvents, contamination can be attributed to the high frequency of detection of these compounds in groundwater and their high persistence and toxicity [1]. The remediation of groundwater contaminated by chlorinated solvents, harmful compounds classified as carcinogenic by the Italian legislature [2], has been usually carried out using energy-intensive technologies (e.g., pump-and-treat or chemical treatment) [3]. “In-Situ” biological technologies have been introduced in recent years as an alternative to traditional methods [4,5,6]. Particularly, highly chlorinated compounds can be used as electron acceptors; thus, reduced in anaerobic conditions and contemporarily, an organic substrate is oxidized (e.g., lactate, butyrate, or other fermentable substrates), providing the real electron donor (H_2_) [2]. Indeed, the metabolism of specific microorganisms (e.g., *Dehalococcoides mccartyi* (*Dhc*)) converts the parental compounds to more acceptable molecules, i.e., ethylene, through the biological reductive dechlorination (RD) reaction. Several parameters must be considered when designing a full-scale intervention by exploiting the microorganisms naturally present on the site; therefore, bioremediation interventions are considered knowledge-intensive technologies, as the preliminary characterization and evaluation of the overall kinetics are fundamental for the achievement of the remediation objectives [7].

It is well-known that the kinetics of degradation decreases with the chlorination degree due to a corresponding decrease in the efficiency, and because a limited group of organohalide-respiring bacteria can perform it [8,9]. Because of the possible formation and accumulation of harmful intermediates (notably the vinyl chloride (VC), which is a cancerogenic and more volatile compound [10]), the addition of an electron donor alone to enhance the RD is not sufficient to achieve the remediation objectives [11].

To face biological degradation limits, the main innovative strategies provide the use of fermentable biopolymers [12,13], the direct push of activated carbon-based amendments products [14,15,16], or the realization of systems that bypass the addition of the electron donors, replacing them with electrochemical potential (e.g., bioelectrochemical systems) [17,18,19].

In compliance with sustainability and circular economy principles, the scientific community shows great interest in alternative materials such as long-lasting electron donors as possible growth support for biofilm and as adsorbents [20,21,22]. Given these premises, it is reasonable to pay attention to biomaterials and evaluate their effectiveness in the context of environmental remediation, assessing whether they can stimulate the biodegradation process on-site [23].

In this regard, our work aims to use a biopolymer belonging to the class of polyhydroxyalkanoates (PHA), which are biodegradable polyesters naturally synthesized by microorganisms [24], combined with a carbonaceous material from biomass recovery, i.e., biochar, which has adsorbent properties [20,25,26]. The polyhydroxy butyrate (PHB) is produced by a biotechnological process through intracellular storage under unbalanced conditions of growth [27]. In recent years, the use of PHB in groundwater remediation contexts has been evaluated with good results in recent field studies [11]. Like all the PHA, PHB is completely biodegradable and, consequently, it is a source of organic acids and hydrogen [13]. On the other hand, the biochar can be advantageously obtained from biomass residues with adsorption characteristics that depend on the applied thermal treatment [28,29,30].

With these objectives, we have performed a PHB-biochar reactor in a mini-pilot scale (11.3 L of geometric volume), where the coupled adsorption and biodegradation (CAB) mechanism on a biochar pine wood (PWB) was supported by the PHB fermentation [31]. The CAB process on PWB has been already investigated for the removal of TCE from aqueous solutions under lactate-feeding [32]. In this case, however, we chose to build the reactor in a particular multi-zone reactive configuration, including the use of PHB as a controlled release electron donor [31]. In situ, slow fermentation of the substrates should allow the release of a low concentration of hydrogen. Under low H_2_ partial pressure, dechlorinating microorganisms should be favored for other H_2_-using microorganisms [5].

Previous studies have demonstrated PHB fermentability [33] and its effectiveness when combined with zero-valent iron (ZVI) for remediation applications [12,34]. Recent studies recommend biochar to stimulate anaerobic digestion [35,36,37], but there is a lack of studies concerning possible environmental applications and evaluating the synergistic effect of biochar and specific dechlorinating bacteria.

In this work, the reactive materials are presented with the design and the startup phase of the PHB-biochar reactor (fifty days of continuous feeding). The research pinpoints the possibility of using biochar and a PHA as a specific *Dhc* support to treat high concentrations of TCE (close to 80 mg/day).

## 2. Materials and Methods

### 2.1. Biochar from Pine Wood (PWB)

The biochar was derived from pine wood wastes gasified at about 850 °C (Plößberg bei Tirschenreuth, Germany). Details of its production and morphological characteristics are reported by Silvani et al. (2017) and Rossi et al. (2021) [38,39]. Previous works have shed light on its adsorption effectiveness towards several pollutants thanks to its high specific surface area (343 ± 2 m^2^/g) and microporosity (224 m^2^/g) [38,40]. Focusing on chlorinated pollutants, our group has already verified the PWB’s TCE removal capacity [39]. Moreover, the positive effect of the coupled adsorption and biodegradation (CAB) on the PWB was also investigated to verify if the PWB can support the *Dhc* activity [32].

### 2.2. The Polyhydroxybutyrate (PHB)

A commercial PHB was used as an electron donor source.

**Figure 1:**
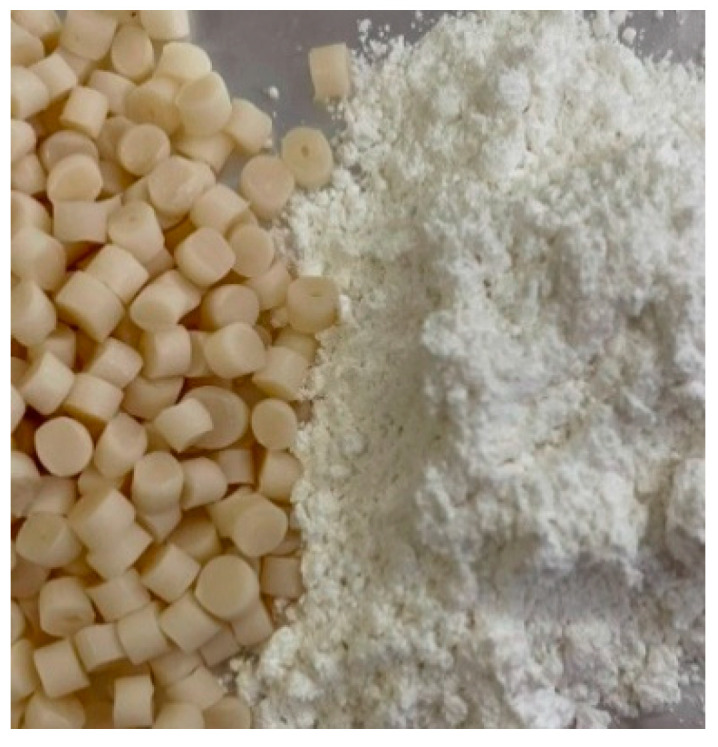
Two forms of Polyhydroxybutyrate (PHB): pellets (on the left) and fine powder (on the right).

To allow a long-lasting release of electron donors, two forms (see Figure 1) were mixed with soil (4% *w*/*w*): the powder form to ensure quick fermentation (since no fermentative biomass has been added in the fermentative zone), and the pellet form to guarantee a higher and consistent duration [22]. As mentioned above, previous studies have demonstrated the effectiveness of PHB in sustaining the RD reaction mediated by *Dhc* [41], with very interesting results in a field-scale process specifically focused on remediation applications [6,42].

### 2.3. Dehalococcoides (Dhc) Enriched Biomass

The dechlorinating enriched culture was fed with TCE and lactate at a sludge retention time of 30 days. Its characterization is reported in Rossi et al. (2022) [32], and analytical methods are described in Ritalahti et al. (2006) and Matturro et al. (2013) [43,44]. Specifically, fermentative species and organohalide-respiring microorganisms mainly populated the culture (45% of *Clostridium sensu stricto 7* and 34.5% of *Dhc mccartyi*). More specifically, the 16S rRNA accounted for 4 × 10^8^ gene copies/L, expressing *tceA*, *bvcA* (1.06 × 10^8^ and 9.26 × 10^5^ gene copies/L, respectively), and *vcrA,* with an abundance of ≤1 × 10^3^ gene copies/L [32]. The addition of enriched culture in the system had the aim to simulate a natural site condition or a bio-augmentation strategy approach [22,45].

### 2.4. Design of the PHB-PWB Reactor

**Figure 2:**
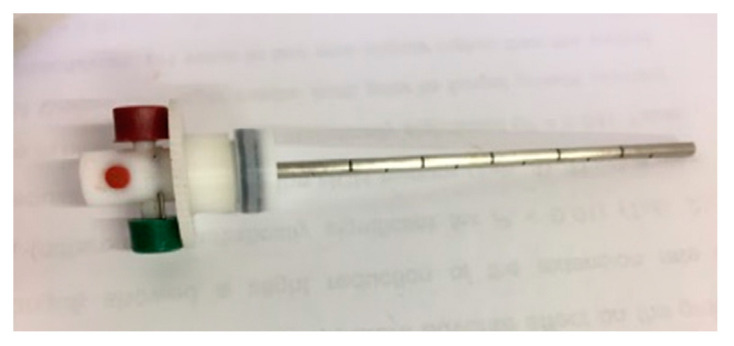
Sampling ports located on the 13 lateral gates.

**Figure 3:**
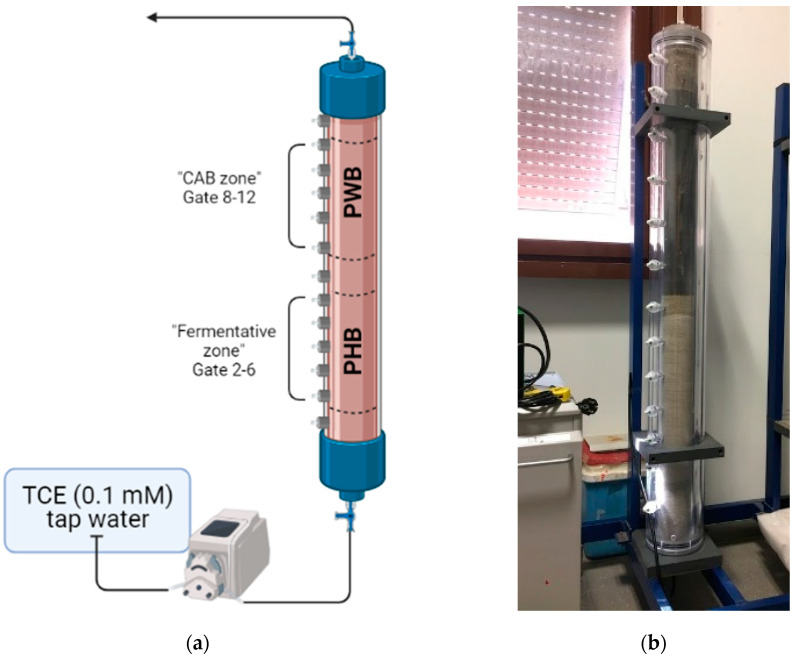
(**a**) Schematic setup; (**b**) a photograph of the Polyhydroxybutyrate (PHB)-biochar reactor.

The reactor consisted of a polymethylmethacrylate (PMMA) column. The central body was 144 cm long, and the inner diameter was 10 cm. The column was equipped on one side with 13 side gates wherein sampling ports with mininert™ valves (VICI, Schenkon, Switzerland) were located (Figure 2). The reactor was designed to accommodate two different reactive zones, hereinafter called the “fermentative zone” and the “CAB zone”. Figure 3a shows the schematic setup. Before adding the reactive materials, the soil was washed and sieved by wet screening using the Vibrating Screen Retsch AS 200 (Haan, Germany). The particle size distribution was divided as follows: 45.4% with d > 2 mm; 19.7% d > 1 mm; 14.9% d > 500 µm; 18.1% d > 125 µm, and 1.8% d > 63 µm. To simulate sandy soil, fractions were mixed in this percentage: 44% > 2 mm + 20% > 1 mm + 36% < 1 mm. The total mass sifted and used for the filling material was close to 20 kg.

Consequently, a layer of soil was left in correspondence with gate number 1, then the “fermentative zone” was realized from door number 2 to door number 6 for a total of 50 cm, where PHB was distributed at 4% *w*/*w* of the total filling weight in the form of fine powder (50 g) and pellets (200 g). A layer of soil has been left to separate the second reactive zone (corresponding to gate 7). Finally, the “CAB zone” consisted of 4% *w*/*w* of PWB (300 g) mixed with soil and then distributed from gate number 8 to 12, for a total of 57 cm (see Figure 3b). Gate number 13 indicates the last gate before the exit (only soil), and a three-way valve was installed to sample the effluent (defined as port number 14).

To saturate the filling material and verify that there were no leaks of the liquid phase, the column was filled with distilled water. Once the flow rate reached a stable value (around 6 L/day), a tracer test was carried out by using a NaCl solution (0.02 M) and measuring the conductivity over time using a Handylab^®^ 330 (SI-analytics, Weilheim, Germany) [46]. Data were elaborated with SigmaPlot 12 to obtain the effective residential time and to calculate the porosity [39]. After at least two days of washing, the reactor was filled with a solution for biomass growth (details of the mineral medium composition are reported elsewhere [46]). Thereafter, only the CAB zone was inoculated with 1 L of the *Dhc* enriched biomass. To ensure biofilm acclimation, the reactor was closed for 48 h. The temperature of the reactor was maintained at 28–30 °C by an external jacket.

### 2.5. Feeding Solution and Working Conditions

The feeding solution was prepared by flushing the tap water with a gas mixture of N_2_/CO_2_ (70/30% *v*/*v*) to create the anoxic condition, then collecting the solution into a 25 L gas-tight auto collapsing Tedlar bag^®^ (Supelco, Cerritos, CA, USA) to avoid the headspace formation. TCE (ACS ≥ 99.5%, Sigma-Aldrich^®^, St. Louis, MO, USA), was spiked inside the bag through a septum (to achieve a final concentration of 0.1 mM, i.e., ≅14 mg/L). The feeding solution flowed from the bottom of the column with a VELP (Usmate Velate, MB, Italy) peristaltic pump. The effluent was also collected in a Tedlar bag^®^ to ensure that no release of toxic compounds into the environment occurred.

In this work, the first 50 days (from September to the end of October 2020) of operation are reported. The theoretical operating conditions are summarized in Table 1. However, the effective flow rate was daily calculated since the intrinsic fluctuations of the adopted pumping system must be considered. The results will therefore show the average values over the investigated period. Similarly, the actual TCE incoming (TCE in) concentration will be determined by sampling when changing each feeding solution (analytical methods reported in Section 2.6.2).

The CAHs and organic acids monitoring started after almost two hydraulic retention times (HRT) of continuous feeding. On the other hand, the inlet and the outlet were sampled every day, whereas the profiles were performed once a week, both for the CAHs and organic acids determination. Periodically liquid samples were also collected to determine sulfate concentration because SO_4_^2−^ is a possible electron acceptor that competes with TCE [47,48,49,50].

### 2.6. Analytical Methods

#### 2.6.1. PHB Characterization

Thermogravimetric analyses (TGA) and differential scanning calorimetry (DSC) were carried out on PHB powder and pellets to verify possible correlations between the polymer form and the biodegradation process. Since the PHB pellets did not dissolve completely in chloroform, it has not been possible to evaluate the sample purity by the conventional gas chromatographic method. Moreover, the presence of additives could interfere with this analysis. Therefore, the purity, as well as the thermal stability, of PHB samples were investigated by thermogravimetric analysis (TGA), using a Mettler TG 50 thermobalance equipped with a Mettler TC 10 A processor.

All measurements were carried out under nitrogen flow by heating about 7 mg of sample from 25 °C to 500 °C at 10 °C/min.

The thermal properties were characterized by a differential scanning calorimetry (DSC) Mettler Toledo DSC 822^e^. The PHB samples were subjected to the following temperature program: the first heating from 25 °C to 190 °C at a 10 °C/min, used to erase the polymer’s previous thermal history; a cooling from 190 °C to −70 °C at 30 °C/min; and, finally, a second heating up to 190 °C at 10 °C/min. The sample calorimetric crystallinity ($X_{c}$) was evaluated by the equation:

(1) $$X_{c}=\frac{\Delta H_{m}}{w\Delta H_{m}^{0}}$$

where $\Delta H_{m}$ is the measured melting enthalpy, $\Delta H_{m}^{0}=146$ J/g the enthalpy of fusion of 100% crystalline PHB [51], and *w* the sample purity.

#### 2.6.2. Determination of the CAHs

To verify the correct input TCE concentration, the sample was taken through the septum of the Tedlar bag^®^ with a micro-glass syringe (Hamilton, Switzerland). On the other hand, the determination of the chlorinated intermediate was performed by taking 1 mL of the liquid phase from each side door with a plastic syringe and injected directly into a hermetically closed vial. The analysis was conducted on a gas chromatograph (GC) Dani Master (DANI Instruments, Contone, Switzerland) equipped with a flame ionization detector (FID), a capillary column (30 m × 0.53 mm ID × 3 um, TRB 624), and a DANI 86.50 headspace auto-sampler (Milan, Italy). The headspace analysis program was performed with an oven temperature of 80 °C, manifold at 120 °C, with the transfer line temperature at 180 °C. The vial was shaken softly for 1 min. To follow, the GC carrier gas (flow 10 mL/min) was He, the injector temperature was 180 °C (split injection 1:2), and the FID detector was at 200 °C with air, N_2_, and H_2_ (flows 240, 25, 60 mL/min). To analyze TCE, cis-DCE, and VC, the oven temperature was programmed as follows: 60 °C for 3 min, 30 °C/min to 120 °C for 5 min.

#### 2.6.3. Determination of Organic Acids

The fermentation process was monitored by sampling about 2.5 mL of the liquid phase from the reactor for the analysis of organic acids (indeed, acetic acid and butyric acid are produced by the fermentation of PHB) [33]. For the analysis, the samples were filtered (0.22 µm porosity) and analyzed using gas chromatography [13]. Briefly, 100 µL of oxalic acid (0.33 M) was added to 1 mL of the filtered sample, and 1 µL of the solution was injected into a GC Dani Master (Milano, Italy) equipped with a 2 m × 2 mm glass packed column in a Carbopack. The carrier gas flowed at 25 mL/min; the injector temperature was 200 °C and the oven temperature was 200 °C; the flame ionization detector (FID) temperature was 200 °C.

#### 2.6.4. Determination of Sulfates

Sulfates were determined with ionic chromatography “Dionex ICS-1000 IC” (Sunnyvale, CA, USA) ICS-1000 IC with a conductivity cell detector equipped with a Dionex AS-40 Auto sampler. Pre-column Dionex IonPac™ AG14 (4 mm × 50 mm) and a Dionex IonPac™ AS14 IC Column with suppressor AESR 500 4 mm (Thermo Fisher Scientific Chelmsford, MA, USA) were used. The eluent phase was prepared with 3.5 mM Na_2_CO_3_ and 1.0 mM NaHCO_3_ solutions with 1.2 mL/min as the flow rate. The calibration curve was realized from 5 to 100 mg/L of the corresponding salt Na_2_SO_4_, Sigma-Aldrich^®^ (St. Louis, MO, USA).

## 3. Results and Discussion

### 3.1. PHB Characterization

**Figure 4:**
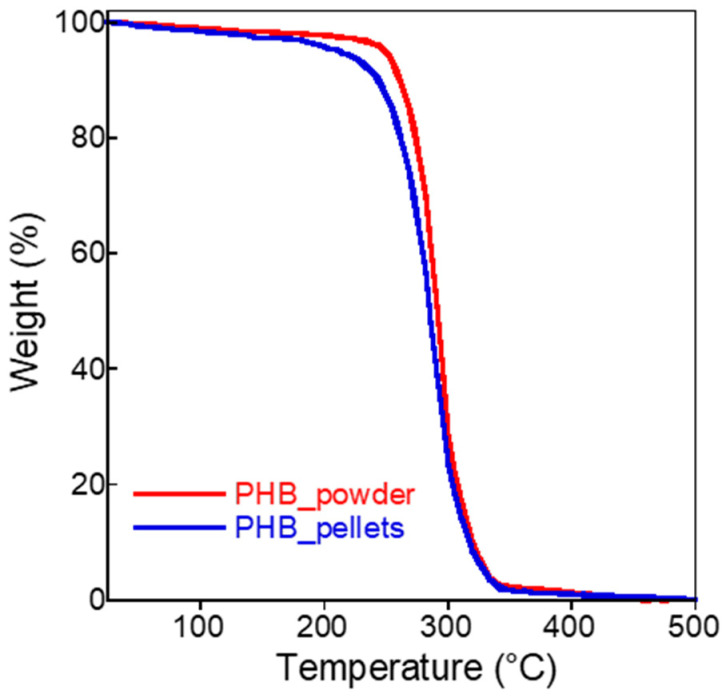
TGA curves of PHB powder and pellets samples.

TGA was employed to estimate the thermal stability and purity of PHB in powder and pellet forms. As it can be seen from the TGA curves in Figure 4, the samples showed slightly different behavior. The sample obtained from the pellets displayed a continuous weight loss of about 4% *w*/*w* up to about 195 °C, the temperature at which the characteristic PHB decomposition takes place (from 95% *w*/*w* to a residual weight of 2% *w*/*w*) [52]. Thus, the purity of about 93% *w/w* can be estimated. On the other hand, the PHB powder sample was nearly stable up to 240 °C (weight loss 2% *w/w*) and then decomposed up to a residual 2% *w*/*w*, showing a purity of about 96% *w*/*w*. The difference between the two samples was caused by the presence of additives in the pellets, e.g., plasticizer for the extrusion process. Indeed, the presence of plasticizer in commercial PHB-pellets, specifically the tri-*n*-butyl citrate (TBC), is reported in the literature [53]. The temperature of the maximum decomposition rate (T_d_^max^), reported in Table 2, shows the slightly higher stability of the powder sample (T_d_^max^ = 290 °C), presumably because of its higher crystallinity compared to that of the PHB pellets (T_d_^max^ = 285 °C).

**Figure 5:**
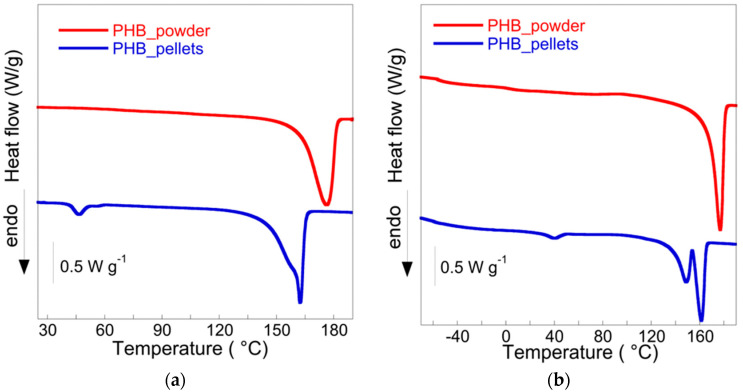
(**a**) First heating scan and (**b**) second heating scan of PHB powder and pellets.

The DSC thermograms of powder and pellets recorded in the first and second heating scans are displayed in Figure 5. The melting temperatures and enthalpies, as well as crystallinity, are reported in Table 2. In the first and the second heating scan, PHB pellets showed a small endotherm peak at about 40 °C, related to the enthalpy recovery of the rigid amorphous fraction [54], and, between 135 and 170 °C, they also showed the double melting process due to the melting of imperfect or thin crystallites, followed by their subsequent recrystallization as thickened crystals and re-melting. Moreover, because of the presence of the plasticizer, the melting temperature and the enthalpy were lower than those of the PHB powder, which is characterized by a higher melting enthalpy (97 J/g for the powder compared to 69 J/g for the pellets) and temperature as well as crystallinity (Table 2).

### 3.2. Tracer Test on the PHB-Biochar Reactor

**Figure 6:**
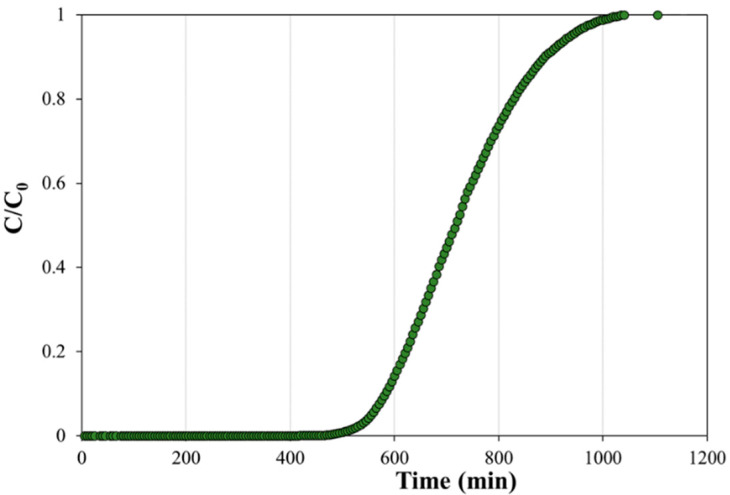
Tracer test of PHB-Biochar column.

The F curve showed the typical trend of a plug flow reactor (Figure 6) with no stagnant zones of preferential flow paths. A slightly dispersed sigmoid was possibly caused by the non-constant grain size along with the reactor profile, evidently due to the presence of the reactive materials. The resulting HRT was about two days, whereas the effective residence time was equal to 716.7 ± 0.8 min, corresponding to an effective linear velocity of 274.6 cm/day. The empty volume (from the multiplication of the effective residence time and flow rate) was about 3146 cm^3^, with a porosity of 28%.

### 3.3. Results from the CAHs Monitoring

**Figure 7:**
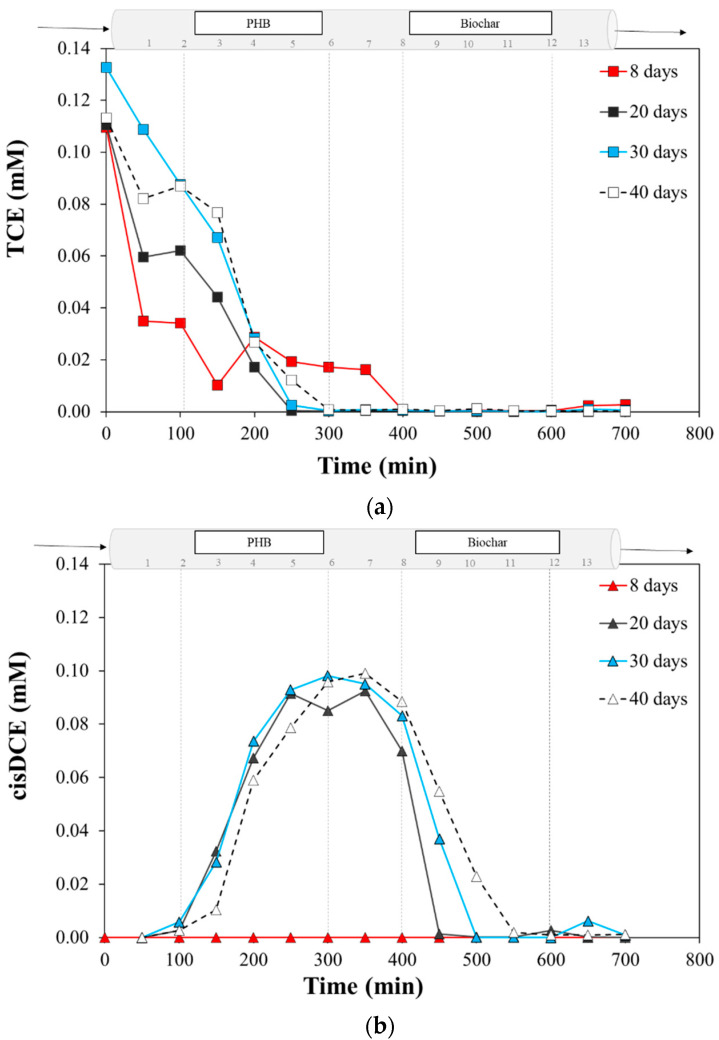

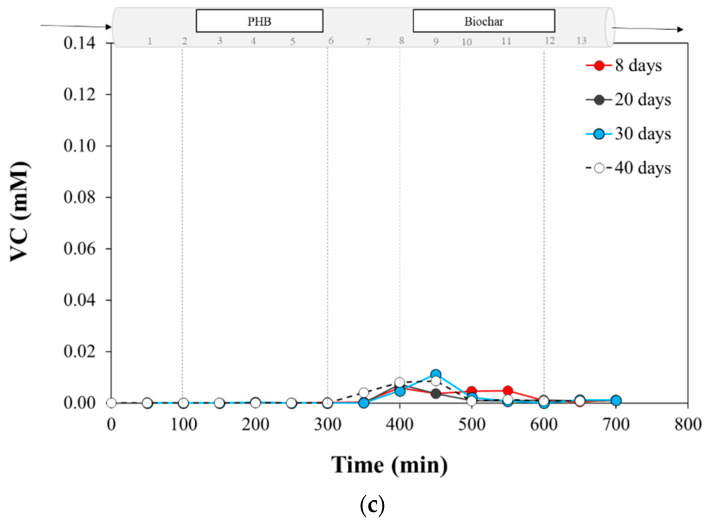
Axial profile as a function of time of (**a**) TCE; (**b**) cis-DCE and (**c**) VC at different operation days.

The following panels show the axial profiles of TCE and daughter products (cis-DCE and VC) as a function of time revealed at 8, 20, 30, and 40 days from the beginning of the test (Figure 7a–c). These moments have been chosen because they were representative of a stable reactor behavior, although every week the samples were collected, analyzed, and stored at −20 °C. It is noteworthy that the concentration of TCE at gate number 1 has been lower than TCE in, but no daughter products were revealed. The reason is leakage of TCE due to the sampling procedure, as the feed concentration was obtained by taking the sample with a glass syringe directly from the Tedlar bag^®^ septum. On the other hand, sampling from the side doors of the reactor has been performed using a plastic syringe, which requires attention and precision (procedure described in Section 2.6.2). Additionally, the presence of tubes and connectors from the pump may result in TCE losses. Nevertheless, observing the trend of the incoming contaminant only, the first profile (8 days) shows that the concentration of TCE was constant in the “fermentative zone”, whereas, dropped from gate 8, it was quickly removed from the aqueous phase by the adsorption mechanism on PWB [39]. On the other hand, very small concentrations of VC were detected (possibly a residual concentration of the inoculum activity). As the days of operation increased, the subsequent profiles show that the kinetics of TCE removal increased, as the TCE was already below the detection limits at gates number 5–6 (250–300 min). Moreover, the presence of TCE reduction products should be considered as confirmation of TCE removal due to biological activity [8]. Indeed, Figure 7b shows the evident formation of cis-DCE, from 20 days of work, in correspondence with the degradation of the TCE. In correspondence with the minimum of TCE, the cis-DCE concentrations achieved were 0.092, 0.098, and 0.099 mM at 20, 30, and 40 days, respectively, suggesting a quantitative degradation of TCE in the di-substituted chlorinated by-product.

Similarly, from gate number 8 a decrease in the concentration of cis-DCE can also be observed in correspondence with the PWB presence. Otherwise, the same sampling profiles (8, 20, 30, 40 days) do not show an evident formation of VC (Figure 7c). This behavior is in agreement with the evidence of a previous study on the CAB process on PWB supported by lactate, where a breakthrough of the RD products was observed after one month of continuous feeding [32]. However, it is important to highlight that, at the same time in the reactor (i.e., at the same sampling gate), an increase in dissolved cis-DCE was observed as the days increased. This behavior can be addressed to a different PWB affinity for less chlorinated compounds [32]. Since the previous test also showed that the biodegradation efficiency depended on the residence time [32], only the monitoring of the next few months will be able to bring to light the progress of the RD reaction with consequent conversion to ethylene.

**Figure 8:**
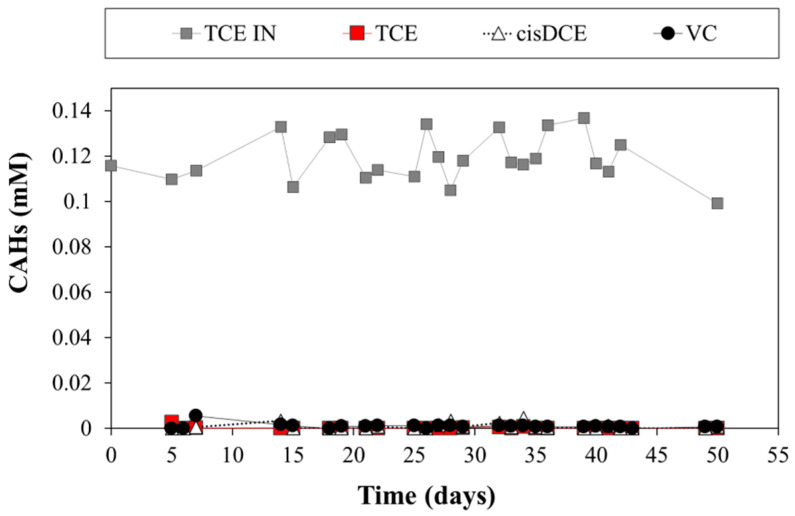
CAHs concentration in the influent and effluent of the reactor during the monitored period.

Overall, during 50 days of monitoring, the CAB mechanism occurred in the reactor as well, as it can be confirmed that the adsorption was also the dominant mechanism in less chlorinated products; indeed, no chlorinated species were detected in the effluent (Figure 8). The previous test [32] (bench-scale 23 cm × 2.6 cm of a fixed-bed column) showed that the biofilm on PWB removed TCE quantitively and continuously for eight months (3–4 mg TCE/day), never achieving the characteristic breakthrough point of the adsorption mechanism alone. However, after one month of operation, cis-DCE and VC were detected within the effluent, indicating that these intermediates had partially broken through the PWB. In that work, an abundance of 9.2 × 10^5^ *Dhc mccartyi* 16S rRNA (gene copies/g) was detected in the PWB-reactor, particularly expressing the RD-essential genes *bvcA* and *vcrA* (1.28 × 10^5^ and 8.01 × 10^3^ gene copies/g, respectively) [32]. In that study, lactate was used as a simple and traditional electron donor source. These results were in agreement with other researchers, reporting that thanks to the combined strategy of adsorption and biodegradation, the life span of the adsorption material could be prolonged [55,56]. In this study, TCE quick removal already in the PHB-zone, where no *Dhc* enriched inoculum was injected, made us hypothesize that the *Dhc* enriched culture diffused in the PHB-zone, or that the colonization of a mixed bacterial community able to degrade TCE occurred [57]. For this reason, samples were taken for future microbiological characterization analyses.

### 3.4. Results from the Organic Acids Monitoring

**Figure 9:**
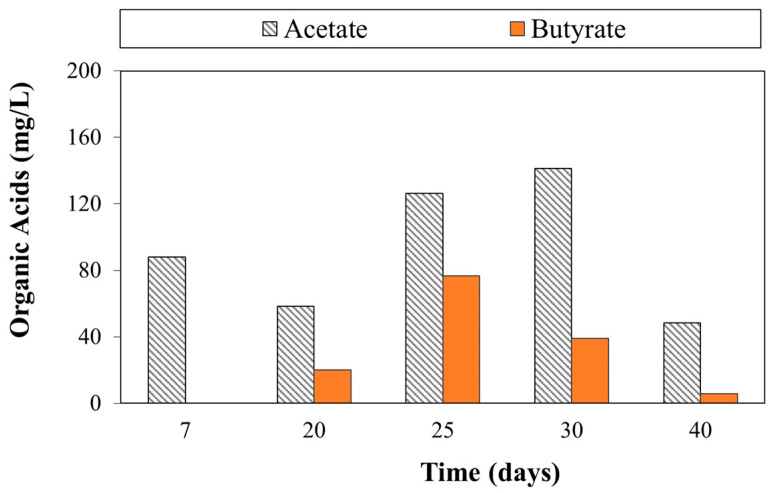
Organic acids concentration in the effluent at different operation days.

Organic acid monitoring confirmed that PHB fermentation had been occurring from the beginning of the test (Figure 9) and continuously for the investigated period. There might be two explanations: either the fermentative bacteria had migrated from the CAB zone to the electron donor source or ubiquitous microorganisms were present in the tap water [12]. The biological degradation reaction of PHB has already been characterized in earlier studies and could be divided into two steps, hence the butyrate results from the first hydrolysis of PHB in hydroxybutyrate (HB) and the acetate (and hydrogen) results from the following β-oxidation of HB [33]. Butyrate and acetate production increased along the reactor, and an accumulation of acetate in the last gates (from a mole of butyrate, 2 moles of acetate are also released) was observed.

The trend of organic acids in the outlet at 7, 20, 25, 30, 40, and 50 days clearly shows an initial burst in the first month of operation, with a maximum concentration produced at 30 days, with 180 mg/L derived from the sum of acetate and butyrate. As mentioned previously, this finding confirms the higher biodegradability of the PHB-powder. On the other hand, PHB pellets need more time to undergo biological degradation, since a lower specific surface area is available for microorganisms’ biodegradative activity [12]. Considering the 8 and 20 reducing equivalent for acetate and butyrate [33], the reducing equivalents for organic acids formation were 101.8, 62.5, 148.1, 169.1, 54.7, and 43.0 meq/day, calculated at 7, 20, 25, 30, 40, and 50 days, respectively.

Interestingly, no methane production was observed in this first period. Notably, methanogens bacteria can compete for H_2_ consumption and influence the RD reaction [31,57,58]; thus, its formation must be controlled in the future.

### 3.5. Results from Sulfate Monitoring

**Figure 10:**
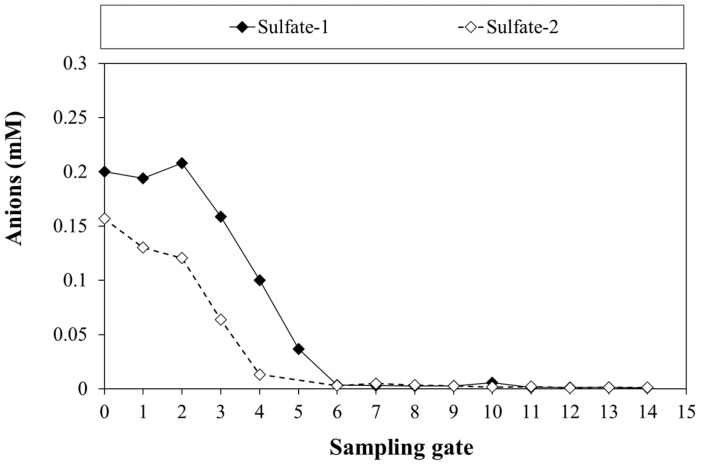
Sulfate concentration monitoring at two different operation days.

It is well known in the literature that sulfate may reduce the kinetics of CAHs degradation (using H_2_ as substrate), and this phenomenon is typically found in chlorinated contaminated sites. Moreover, even though SO_4_^2−^ is quite tolerated by bacteria, its reduction to sulfide could be toxic for microorganisms [49,59]. In this regard, Mao et al. (2017) published an interesting analysis of the effects of the sulfate-reduction on *D. maccartyi*, concluding that sulfide is the toxic compound that inhibits these bacteria [47]. In this context, sulfate levels were monitored, since tap water was used for the feeding solution. The average sulfate concentration in the influent was about 18.8 mg/L (0.2 mM), and two representative patterns are reported as an example (Figure 10). As expected, the sulfate reduction occurred rapidly in the early reactor zone without negatively affecting the degradation of TCE (as described in Section 3.3). Since the behavior was similar over the investigated period, it was decided to reduce the frequency of monitoring the sulfate profile.

## 4. Conclusions

In this work, we have presented two biomaterials coupled to support the biological degradation of a highly diffused chlorinated contaminant. In detail, a commercial PHB and PWB (specifical biochar from pine wood wastes) were combined to enhance the RD in a novel reactor configuration and a mini-pilot scale (11.3 L of reactor volume) to remove TCE from an aqueous solution (close 80 mgTCE/day). The TGA analysis gave an overview of the PHB purity (93 and 96% *w*/*w* for the pellets and the powder form, respectively), and the DSC has confirmed the higher crystallinity of the powder with a higher melting enthalpy and temperature. On the other hand, the presence of additives in the pellets was responsible for the different thermal properties, which is consistent with the literature. Further studies should be focused on the possible release of the additives in the solution and their effect on microorganisms and the quality of the aquifer in field-scale applications.

The PHB-biochar reactor showed the typical trend of a plug flow reactor, with a porosity of 28% and 716.7 ± 0.8 min as an effective residence time. The results show that the CAB process occurs along the reactor already after 20 days. Thanks to the lateral sampling gates, it was possible to observe the quick degradation of TCE (TCE in average 0.12 ± 0.07 mM) in the first fermentative zone, the quantitative cis-DCE formation, and its quick removal by adsorption on the PWB in the second half of the reactor. The first 50 days of monitoring (average of flow rate of about 6.06 ± 0.71 L/day) showed that adsorption was the main mechanism responsible for the absence of chlorinated species in the effluent. Moreover, the organic acid production was observed since the beginning of the test, confirming the prompt biodegradability of the PHB-powder. The following months will shed light on the progress of the RD reaction, on the ability of the PHB-pellets to supply organic acids, and on the adsorption capacity of the PWB.

To the best of our knowledge, this is the first study where PHB and biochar are combined in a mini-pilot configuration for the clean-up of an aqueous solution contaminated by chlorinated compounds.

## Figures and Tables

**Table 1:** Some operating parameters set at the beginning of the test.

PHB-BC Reactor Working Conditions	
Flow rate (L/day)	6
HRT (day)	1.8
TCE in (mM)	0.1
Pore water velocity (cm/day)	76.4

**Table 2:** Thermal properties of PHB powder and pellet samples.

Sample	T_d_^max^ (°C)	1st Heating Scan		2nd Heating Scan			
		T_m_ (°C)	$\Delta H_{m}$ (J/g)	T_g_ (°C)	T_m_ (°C)	$\Delta H_{m}$ (J/g)	$X_{c}$ (wt%)
PHB powder	290	176	89	3	175	97	69
PHB pellets	285	163	78	-	148	69	51

## Data Availability

The data presented in this study are available in the present manuscript.
